# Complete Genome Sequence of *Phodopus sungorus* Papillomavirus Type 1 (PsPV1), a Novel Member of the *Pipapillomavirus* Genus, Isolated from a Siberian Hamster

**DOI:** 10.1128/genomeA.00311-14

**Published:** 2014-04-10

**Authors:** Boštjan J. Kocjan, Lea Hošnjak, Joško Račnik, Marko Zadravec, Mario Poljak

**Affiliations:** a Institute of Microbiology and Immunology, Faculty of Medicine, University of Ljubljana, Ljubljana, Slovenia; b Institute of Poultry Health, Department for Birds, Small Mammals and Reptiles, Veterinary Faculty, University of Ljubljana, Ljubljana, Slovenia

## Abstract

We report the complete genomic sequence of *Phodopus sungorus* papillomavirus type 1 (PsPV1), isolated from an anogenital lesion of a Siberian hamster. PsPV1 is taxonomically classified in the genus *Pipapillomavirus* and is most closely related to *Mesocricetus auratus* papillomavirus 1 (MaPV1).

## GENOME ANNOUNCEMENT

Papillomaviruses (PVs) are a large and diverse group of small double-stranded DNA viruses that are etiologically linked with various benign and malignant lesions of the skin and mucosa. They infect humans and at least 54 other animal species (1). To date, 112 distinct nonhuman papillomavirus types, distributed over 32 different genera, have been genetically characterized. The genus *Pipapillomavirus* currently consists of six PV types: MaPV1, MmuPV1, AsPV1, McPV2, MmiPV1, and RnPV1, isolated from a wide variety of rodent animal species, including the Syrian golden hamster (*Mesocricetus auratus*), house mouse (*Mus musculus*), wood mouse (*Apodemus sylvaticus*), Southern multimammate mouse (*Mastomys coucha*), Eurasian harvest mouse (*Micromys minutus*), and Norway rat (*Rattus norvegicus*), respectively (1). We report here the sequence of a novel *Pipapillomavirus* member, *Phodopus sungorus* papillomavirus type 1 (PsPV1), which was isolated from an anogenital lesion of a Siberian hamster (*Phodopus sungorus*). No PVs have been previously shown to infect this particular animal species.

Partial PsPV1 L1 and E1 gene sequences were initially obtained from the lesion using the hanging-droplet PCR technique with FAP64/FAP6085F (2) and E1Gamma-F/E1Gamma-R primers (3), respectively. A BLAST comparison (http://blast.ncbi.nlm.nih.gov/Blast.cgi) of the partial L1 sequence revealed that PsPV1 clusters to the genus *Pipapillomavirus*. A complete viral genome was amplified using inverse long-range PCR with PsPV1 L1 gene-specific primers and cloned using a TOPO XL PCR cloning kit (Invitrogen, Carlsbad, CA). The complete genomic sequence was determined by the primer walking strategy at Microsynth AG (Balgach, Switzerland) and assembled and characterized using Vector NTI Advance 11 software (Invitrogen). Phylogenetic analysis was performed using a maximum likelihood algorithm (4), and was based on the entire L1 gene sequence of PsPV1 and all currently completely sequenced PVs from the *Gammapapillomavirus*, *Dyoxipapillomavirus*, *Pipapillomavirus*, *Phipapillomavirus*, and *Taupapillomavirus* genera (1).

Sequencing analysis of PsPV1 revealed a genome of 7,630 bp in length, with a G+C content of 47.7%. The genome potentially encodes four early (E1, E2, E6, and E7) and two late open reading frames (ORFs) (L1 and L2), but no E4 and E5 ORFs. The noncoding region of 416 bp is typically positioned between the L1 and E6 ORFs. Phylogenetic analysis confirmed that PsPV1 is a member of the *Pipapillomavirus* genus and is most closely related to *M. auratus* papillomavirus 1 (MaPV1), which was originally obtained from an oral lesion of Syrian hamster (*M. auratus*) (5). With an L1 nucleotide sequence identity of 75.6% to MaPV1, PsPV1 is classified as a new type in the *Pipapillomavirus* genus, species Pi-2.

In conclusion, the present study expands the heterogeneity of the *Pipapillomavirus* genus and suggests a possible etiological role of PsPV1 in the development of anogenital neoplasms in Siberian hamsters, which needs to be investigated further.

### Nucleotide sequence accession number.

The complete genome sequence of PsPV1 has been deposited at DDBJ/ENA/GenBank under the accession no. HG939559.
